# MT-MRI for detection of renal interstitial fibrosis in renovascular disease

**DOI:** 10.1038/s41598-026-60712-6

**Published:** 2026-07-05

**Authors:** YaFei Liu, Dilbar A. Abdurakhimova, Mina Al Saeedi, XiangYang Zhu, Hui Tang, Ailing Xue, Richard G. Frimpong, Sanjay Misra, Gary L. Schwartz, Amir Lerman, Aleksandra Kukla, Lilach O. Lerman

**Affiliations:** 1 Division of Nephrology and Hypertension, Mayo Clinic, 200 First Street SW, Rochester, MN 55905 USA; 2 Department of Nephrology, The first affiliated hospital of ZhengZhou University, 1 JianShe East Avenue, ZhengZhou, 450000 Henan China; 3 Department of Radiology, Mayo Clinic, Rochester, MN 55905 USA; 4 Department of Cardiovascular Diseases, Mayo Clinic, Rochester, MN 55905 USA

**Keywords:** qMT, MTR, BOLD, Fibrosis, Renal vascular disease, Diseases, Medical research, Nephrology

## Abstract

We evaluated kidney fibrosis in renovascular disease (RVD) using quantitative (q) and semi-quantitative magnetization-transfer (MT) magnetic resonance imaging (MRI). Ten RVD patients and 22 healthy volunteers (HVs) were prospectively enrolled. Of HVs, 10 (HV-1) underwent imaging studies whereas plasma samples were collected from 12 others (HV-2). The qMT-derived bound-pool fraction (*f*) was compared to MRI-based MT imaging (MTI)-derived MT ratio (MTR), blood oxygenation-level-dependent (BOLD) R2*, and diffusion-weighted imaging (DWI) apparent diffusion coefficient (ADC). MTR was assessed at offset frequencies of 600 Hz and 1000 Hz, and RVD qMT and MTI on both 1.5T and 3.0T MRI. Additionally, we measured plasma and urinary levels of fibrogenic cytokines and micro-RNAs, and stenotic kidney (STK) perfusion and volume with multidetector computed-tomography (MDCT). At 3.0T-MRI, STK cortex and medulla qMT-*f* were higher in RVD vs. HV-1 (*p* = 0.01, *p* = 0.05, respectively), as was MTR-600 Hz, whereas BOLD-R2* and the DWI ADC were not different. MTR and *f* measured at 1.5T were comparable to those obtained at 3.0T. STK blood flow was decreased vs. the contralateral kidney (CLK) (*p* = 0.033) but plasma and urinary fibrogenic indices were unchanged in RVD vs. HV-2. Both *f* and MTI at 3.0T-MRI may be useful for noninvasive assessment of STK fibrosis, independent of magnetic-field strength. MTI is potentially more sensitive than fibrogenic cytokine levels for detecting mild RVD-related fibrosis changes.

**Supplementary Information:**

The online version contains supplementary material available at https://doi.org/10.1038/s41598-026-60712-6.

## Introduction

The incidence of renovascular disease (RVD) as an etiology for end-stage renal disease (ESRD) has been rising^1^. Revascularization of the stenotic renal artery by percutaneous transluminal renal angioplasty (PTRA) and stenting used to be a common strategy for renovascular hypertension^2^. However, it often fails to restore kidney function in RVD^3^, and in one-third of cases may even accelerate functional decline^4^, highlighting the need to monitor chronic kidney disease (CKD) development and evaluate the integrity of the kidney parenchyma distal to the stenosis. RVD may induce ischemic nephropathy leading to renal fibrosis, the degree and duration of which determine the progression of kidney dysfunction^5^. Therefore, assessing the extent of stenotic kidney (STK) fibrosis in RVD could be invaluable in guiding management decisions.

Noninvasive strategies to detect and quantify renal fibrosis remain limited. We previously showed that magnetization transfer imaging (MTI) generates magnetization transfer ratio (MTR) maps and detects STK fibrosis in murine^6^ and swine^7^ models of unilateral RVD at 16.4T and 3.0T, respectively. However, MTI is semi-quantitative and may vary with scanner characteristics. In contrast, quantitative magnetization transfer (qMT) offers assessments of tissue MT properties and macromolecule content (reflecting fibrosis), which are considered more objective and potentially less dependent on scanner characteristics. Yet, the clinical utility of qMT in patients with RVD and its consistency across magnetic-field strengths remain unclear.

Renal fibrosis is traditionally assessed by invasive renal biopsy, which limits its clinical utility. Thus, other noninvasive biomarkers like fibrosis-related miRNAs, cytokines, and levels of extracellular matrix proteins (e.g., collagen) have been applied as indirect indices of renal fibrosis. Collagen-I expression is elevated in injured HK-2 cells^8^ and in diabetic kidney disease -related fibrosis^9^, as is collagen-IV^10^. Furthermore, α1collagen-(I)- and (IV) mRNAs, and tissue inhibitors of metalloproteinase-1 (TIMP-1) mRNAs and proteins, are upregulated in the fibrotic renal cortex of db/db mice^11^. Additionally, exosomal micro-RNAs (miRNAs) cargo found in bodily fluids, like urine and blood, may reflect renal fibrosis^12,13^. However, the ability of these markers to detect mild renal fibrosis in RVD has not been established.

Therefore, we tested the hypothesis that the qMT-derived macromolecule “bound pool fraction” (*f*) could detect development of kidney fibrosis in RVD compared to healthy kidneys, which would correlate with indirect renal fibrosis indices. We further compared the reproducibility of these assessments between 1.5T and 3.0T-MRI to evaluate their robustness across different MRI platforms, which may facilitate clinical translation and multicenter studies.

## Materials and methods

In this prospective, cross-sectional observational study, we compared STK fibrosis assessments obtained using qMT (*f*) and MTR using 1.5T and 3.0T MRI in 10 patients with RVD. Because kidney biopsies were not clinically indicated or available, *f* and MTR were also compared to alternative indices of renal fibrosis, including MRI-based blood oxygenation-level-dependent (BOLD)^14^ and diffusion-weighted imaging (DWI)^15^, as well as to plasma and urinary levels of fibrogenic cytokines and miRNAs in the same patients. To ascertain the hemodynamic significance of the stenosis, we also measured STK perfusion in RVD using multidetector computed-tomography (MDCT). Furthermore, to confirm STK remodeling in RVD we compared MRI measurements to those obtained using 3.0T MRI in healthy volunteers (HV).

This prospective study (ClinicalTrials.gov NCT04508049 Registered 6 August 2020, clinicaltrials.gov/study/NCT04508049) adhered to the principles of the Declaration of Helsinki, was approved by the Institutional Review Board (19-008413) of Mayo Clinic, and all participants provided informed consent. Of 22 HV, 10 (HV-1) underwent 3.0T MRI studies and served as a control group for imaging, whereas blood and urine samples were collected from a separate comparable HV group (HV-2, *n* = 12) to analyze fibrogenic factor levels. Subjects were enrolled according to the inclusion and exclusion criteria (Supplemental file).

### Clinical indices

Clinical and laboratory data were collected from HV-2 participants during research laboratory appointments. In RVD, renal vein and inferior vena cava (IVC) sampling was conducted during catheter placement for MDCT studies. Data on blood pressure, serum creatinine levels, and estimated glomerular filtration rate (eGFR) were retrieved from the medical records.

## Fibrogenic factor protein measurements

To assess fibrogenic activity, we measured plasma levels of pro-collagen-I alpha-1 (R&D, Cat#DPCA00), collagen-IV (Novus, Cat#NBP2-75864), and TIMP-1 (Sigma-Aldrich, RAB0466) in systemic blood in HV-2, as well as in the STK vein, contralateral kidney (CLK) vein, and IVC in RVD. Urinary levels of pro-collagen-I alpha-1 (R&D, Cat#DPCA00), collagen-IV (Novus, Cat#NBP2-75864), and TIMP-1 (Sigma-Aldrich, RAB0466) were also measured.

## Urinary exosome micro-RNAs

Urine exosome RNA isolation kit (Norgen Biotek, Cat#47200) was used to isolate urinary exosomes in HV-2 and RVD. In these we subsequently measured fibrosis-related (miR-214, miR-150, and miR-21) and anti-fibrotic (miR-26a and miR-218) micro-RNAs using reverse transcription polymerase chain reaction (RT-PCR). Urinary levels (all ThermoFisher) of exosome miR-150 (Cat#002637), miR-214 (Cat#002306), miR-21 (Cat#002438), miR-26a (Cat#000405), and miR-218 (Cat#000521) were measured, and Let 7a (Cat#000377) served as the control.

## Imaging procedures

MTI, qMT, BOLD, and DWI non-contrast scans were performed in RVD and HV-1 with a 3.0T MRI Signa scanner (DISCOVERY MR750W, GE). In RVD patients, MTI and qMT were also acquired at a one-day interval using a 1.5T scanner (OPTIMA MR450W, GE, Waukesha, WI). Electrocardiography and blood pressure were continuously monitored throughout scanning.

For specific MTI, qMT, BOLD-MRI, DWI, and MDCT protocols, please see Imaging Procedures in Electronic Supplementary Materials.

### Statistical analysis

GraphPad Prism 10.0.0 was used for statistical analysis. The Shapiro-Wilk test was used to detect deviations from normality. Normally distributed data was summarized using mean ± SD and compared using one-way ANOVA. Non-normally distributed data were summarized as median (quartile1, quartile3) and tested via the Kruskal-Wallis test. *P* ≤ 0.05 was considered statistically significant.

## Results

### Demographic characteristics

There were no significant differences in gender, body mass index (BMI) (*p* = 0.1903), mean arterial pressure (MAP), serum creatinine, or eGFR (*p* = 0.099) between the 10 RVD patients and 10 HV-1 enrolled for imaging analysis, except that RVD were older. CT-angiography demonstrated a hemodynamically significant stenoses (1 bilateral and 9 unilateral) in RVD (Table 1).

**Table 1:** Characteristics of healthy volunteers and patients with renovascular disease.

Parameter	HV-1	HV-2	RVD
Patient number, n	10	12	10
Gender, male/female	3 M/7F	6 M/6F	3 M/7F
Body mass index	24.3 (19.56, 34.73)	25.29 ± 2.40	29.7 (26.65, 35.03) ^#^
Age, Yrs	63.9 ± 6.05	66.08 ± 6.19	70.9 ± 5.74*
Mean arterial pressure, mm/Hg	93.4 ± 7.53	87.85 ± 8.6	93.6 ± 11.97
Serum creatinine, mg/dl	0.95 ± 0.29	0.97 ± 0.21	1.07 ± 0.16
Estimated glomerular filtration rate, ml/min/1.73m^2^	78.13 ± 19.31	77 ± 11.63	64 ± 13.59^#^
Stenosis, %			87.27 ± 6.40

*p ≤ 0.05 vs. HV-1; ^#^*p* ≤ 0.05 vs. HV-2.

HV-1, imaging healthy volunteers; HV-2, plasma healthy volunteers; renovascular disease, RVD.

The sex, age, MAP, and serum creatinine were similar between the RVD and HV-2 subjects enrolled for cytokine analysis, except that RVD had a higher BMI and lower eGFR (Table 1). HV-1 and HV-2 had similar demographic characteristics (Table 1).

## MRI-derived fibrosis at 3.0T and 1.5T-MRI in RVD vs. the CLK and HV-1 subjects

**Fig. 1:**
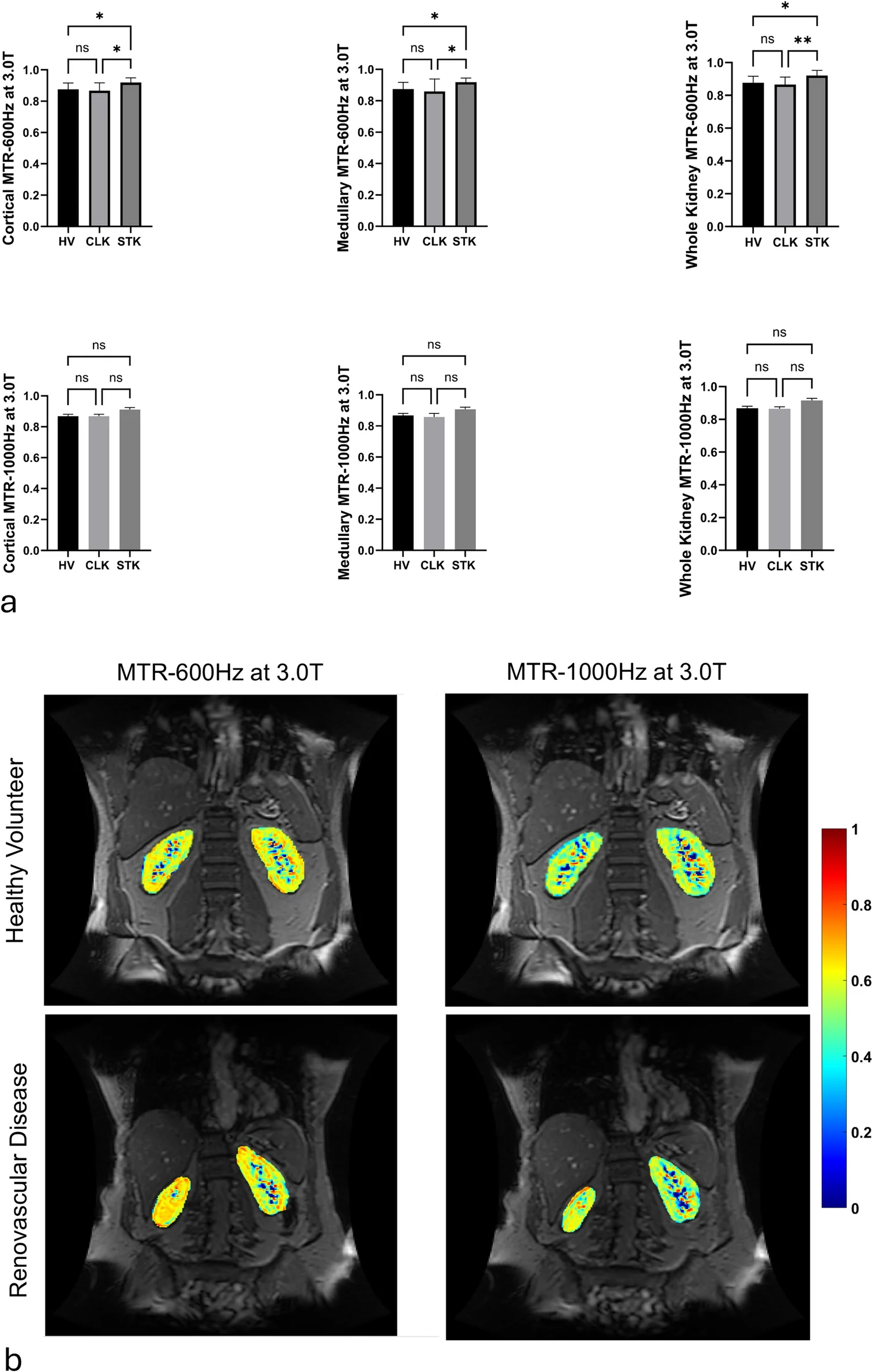
MTR at 3.0T-MRI in stenotic and contralateral kidneys of RVD patients and HV. (**a**) Magnetization transfer ratio (MTR) measured at 600 Hz but not 1000 Hz was higher in the stenotic kidney (STK) cortex, medulla, and whole kidney compared with healthy volunteers (HV)-1 and the contralateral kidney (CLK). (**b**) Representative images of MTR in renovascular disease (RVD) and HV (smaller kidney: STK). *p ≤ 0.05; **p < 0.01; ns, not significant.

**Fig. 2:**
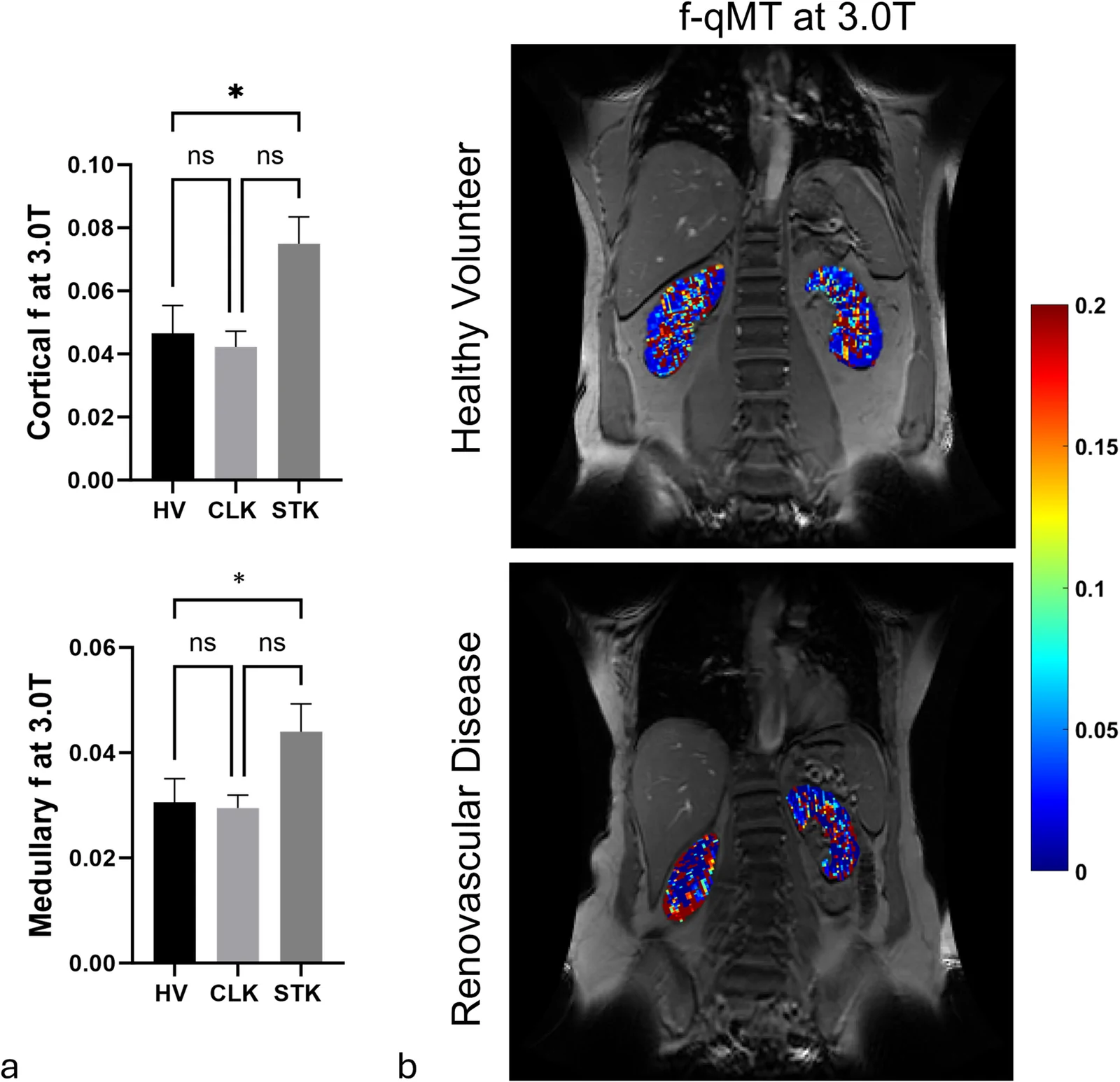
*f* at 3.0T-MRI in RVD patients and HV. (**a**) Quantitative magnetization transfer (qMT)-derived *f* was higher in the stenotic kidney (STK) cortex and medulla compared with healthy volunteers (HV)-1. (**b**) Representative images of *f* in renovascular disease (RVD) and HV (smaller kidney: STK). *p ≤ 0.05; ns, not significant.

**Fig. 3:**
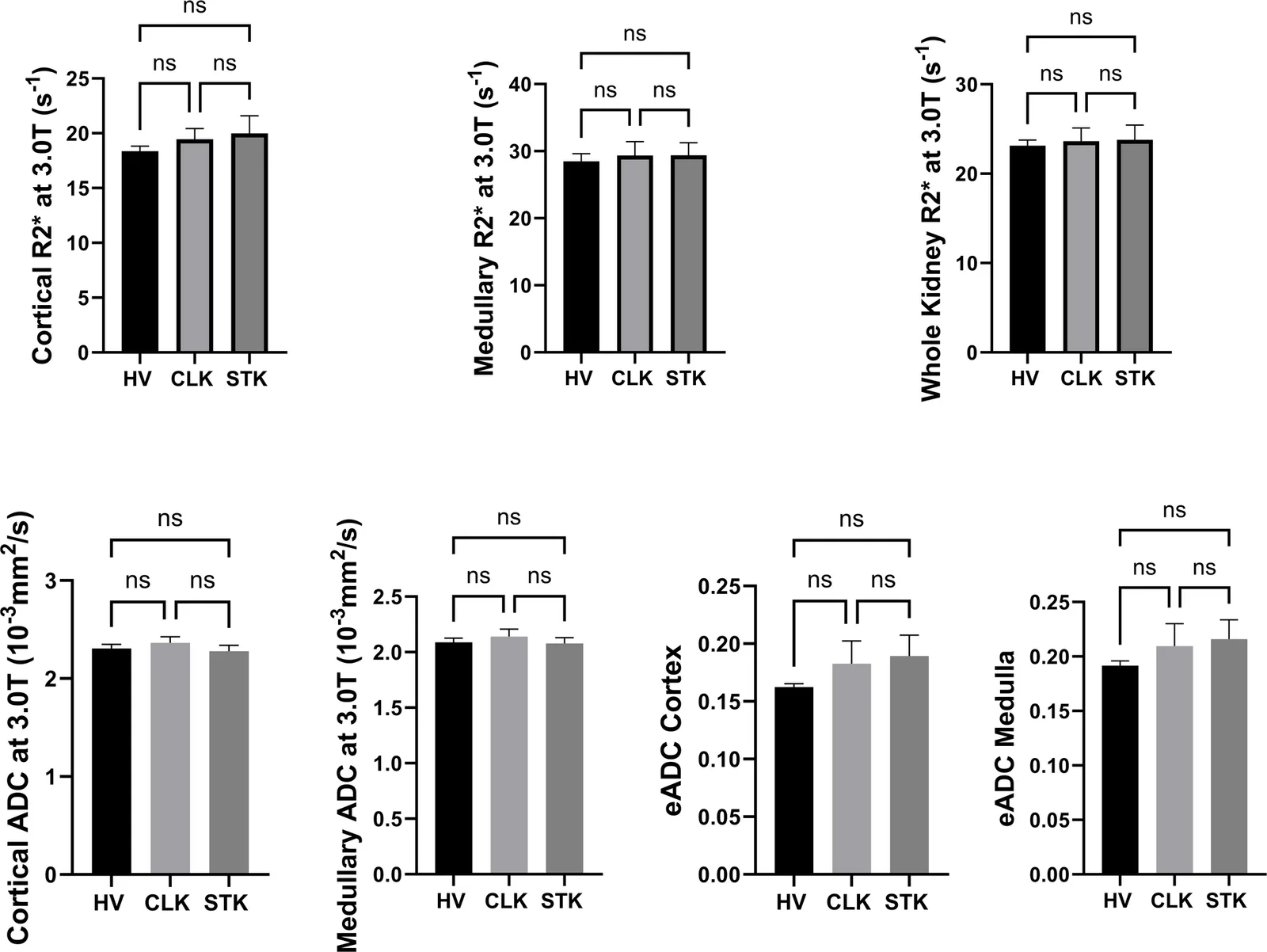
BOLD-R2* and DWI at 3.0T-MRI in contralateral and stenotic kidneys of RVD and HV. Blood oxygenation-level-dependent (BOLD)-R2*, diffusion-weighted imaging (DWI) apparent diffusion coefficient (ADC), and exponential ADC (eADC) were not different among healthy volunteers (HV)-1, contralateral (CLK) and stenotic (STK) kidneys. ns, not significant.

At 3.0T**-**MRI we measured the MTR, qMT, BOLD, and DWI in both RVD and HV-1. MTR measured at 600 Hz was higher in the cortex, medulla, and whole kidney of STK-RVD vs. HV-1, and vs. CLK, whereas 1000 Hz MTR did not distinguish the groups (Fig. 1). The cortical and medullary *f*, reflecting the extent of water-binding large molecules and a surrogate for fibrosis, was also higher in the RVD-STK vs. HV-1 (Fig. 2), and cortical *f* tended to be higher than the CLK as well (*p* = 0.063). In contrast, BOLD-R2* and DWI-derived apparent diffusion coefficient (ADC) and exponential ADC (eADC) were not different among the STK, CLK, and HV-1 (Fig. 3).

**Fig. 4:**
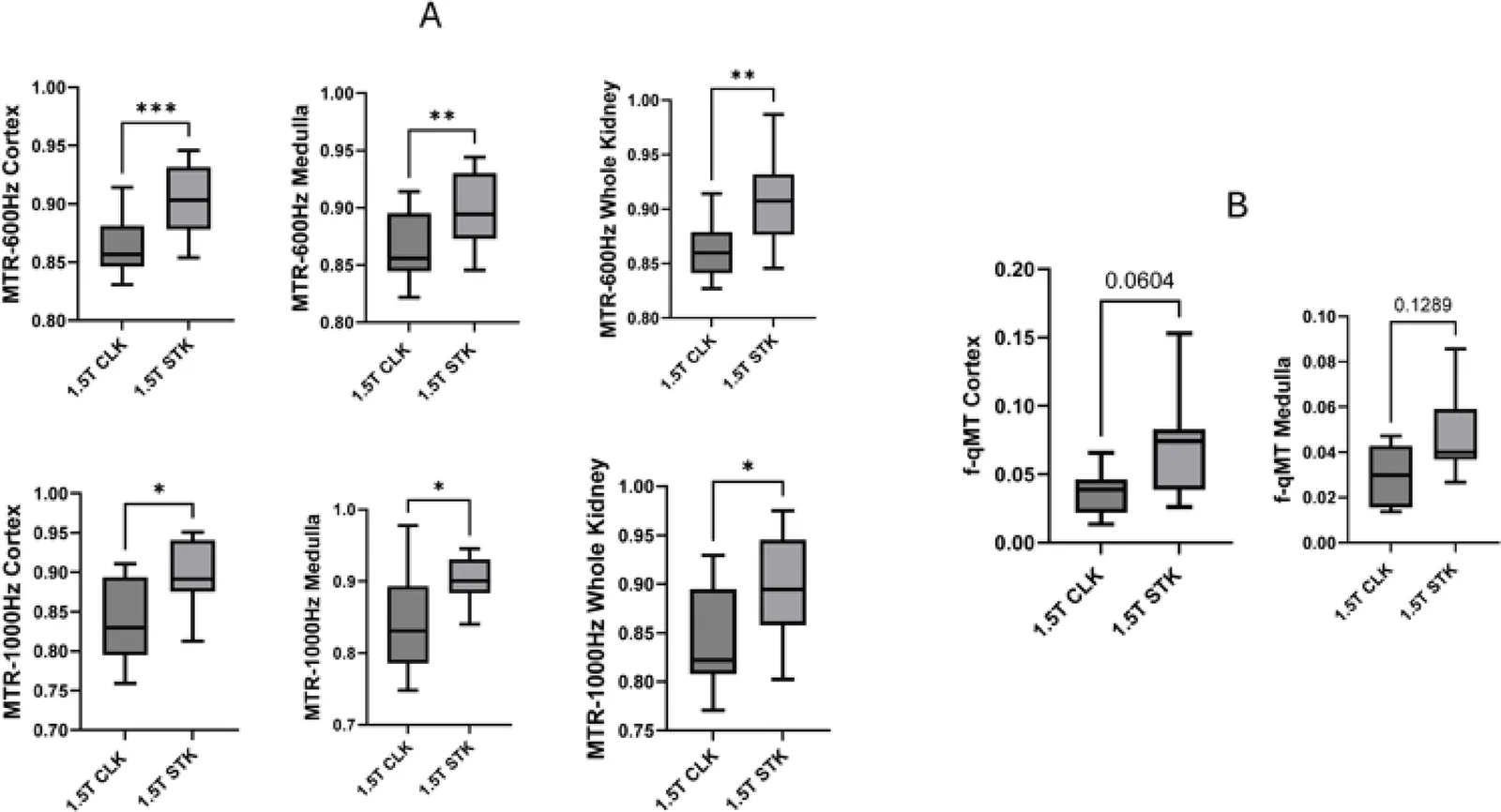
MTR and *f* at 1.5T-MRI in RVD patients. Magnetization transfer ratio (MTR) and *f*-quantitative magnetization transfer (qMT) at 1.5T-MRI in contralateral (CLK) and stenotic (STK) kidneys of renovascular disease (RVD) patients. (**a**) MTR measured at 600 Hz and 1000 Hz were both higher in the STK cortex, medulla, and whole kidney compared with the CLK. (**b**) *f* was not different between the CLK and STK, although it showed a nonsignificant trend toward higher values in the STK cortex. *p ≤ 0.05; **p < 0.01; ***p < 0.0001; ns, not significant.

Similarly to MTR-600 Hz obtained at 3.0T (Fig. 1), MTR measured at 1.5T at both 600 Hz and 1000 Hz were higher in the cortex, medulla, and whole STK vs. the CLK (Fig. 4A). Cortical *f*, but not medullary *f*, also showed a nonsignificant trend toward higher values in the STK vs. CLK at 1.5T (Fig. 4B, *p* = 0.060 and *p* = 0.129, respectively).

### Comparison of qMT and MTI at 1.5T vs. 3.0T MRI in RVD

**Fig. 5:**
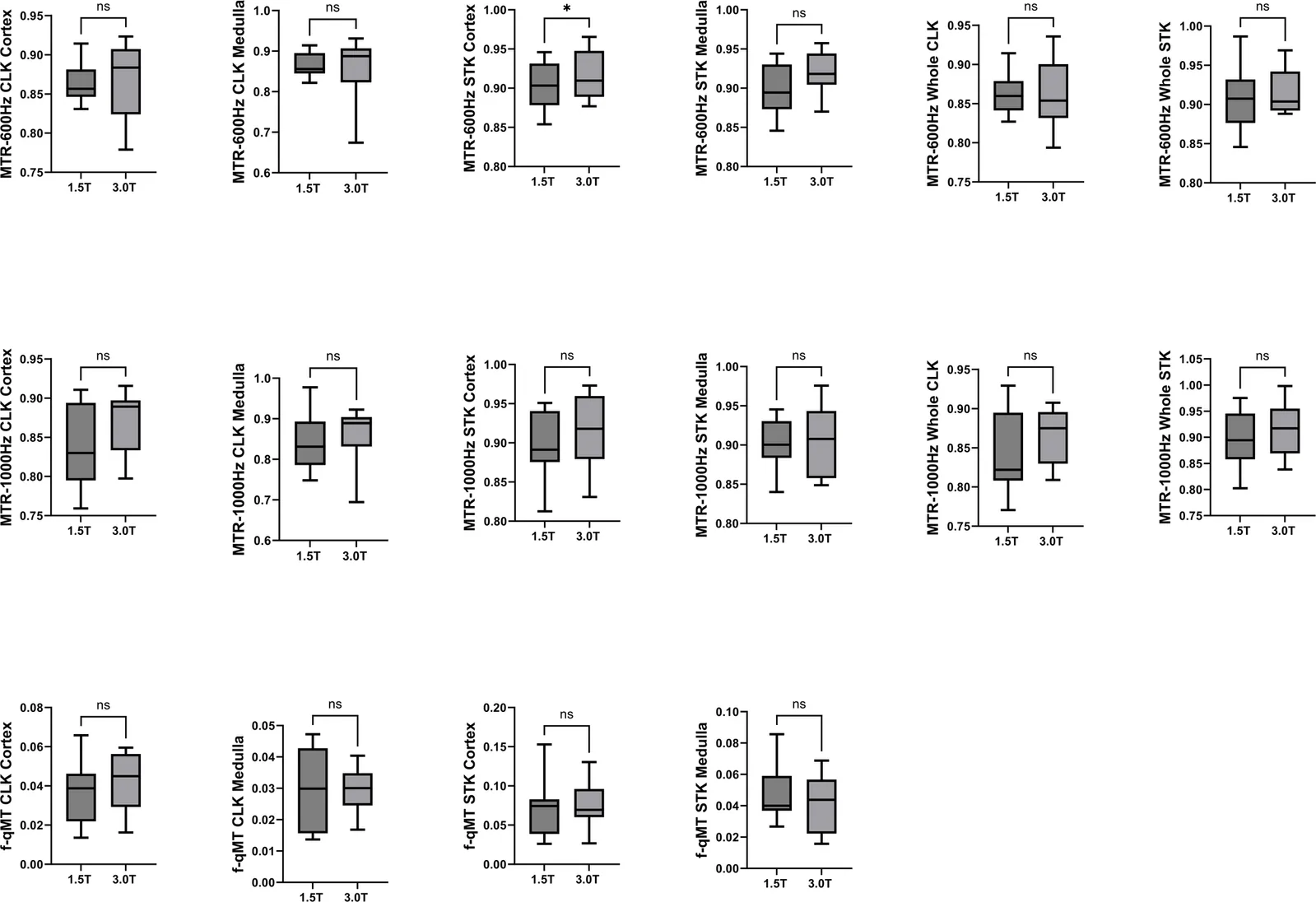
Comparison of 1.5T and 3.0T qMT and MTI in renovascular disease patients (RVD). (a) Cortical, medullary, and whole kidney MTR-600 Hz obtained at 1.5T and 3.0T MRI were not significantly different except STK cortex. (b) Cortical, medullary, and whole kidney MTR-1000 Hz obtained at 1.5T and 3.0T MRI were not significantly different from each other. (c) Cortical and medullary f were not significantly different between the two field-strengths. *p ≤ 0.05; ns, not significant.

MTR of the cortex, medulla, and whole kidney obtained at 600 Hz and 1000 Hz at 1.5T were both comparable to those obtained at 3.0T-MRI, except for STK-cortex at MTR-600 Hz that was lower at 1.5T than at 3.0T-MRI (Fig. 5A-B). *f* in the STK cortex and medulla were not different between 1.5T and 3.0T-MRI (Fig. 5C).

### MDCT measurements in RVD

**Fig. 6:**
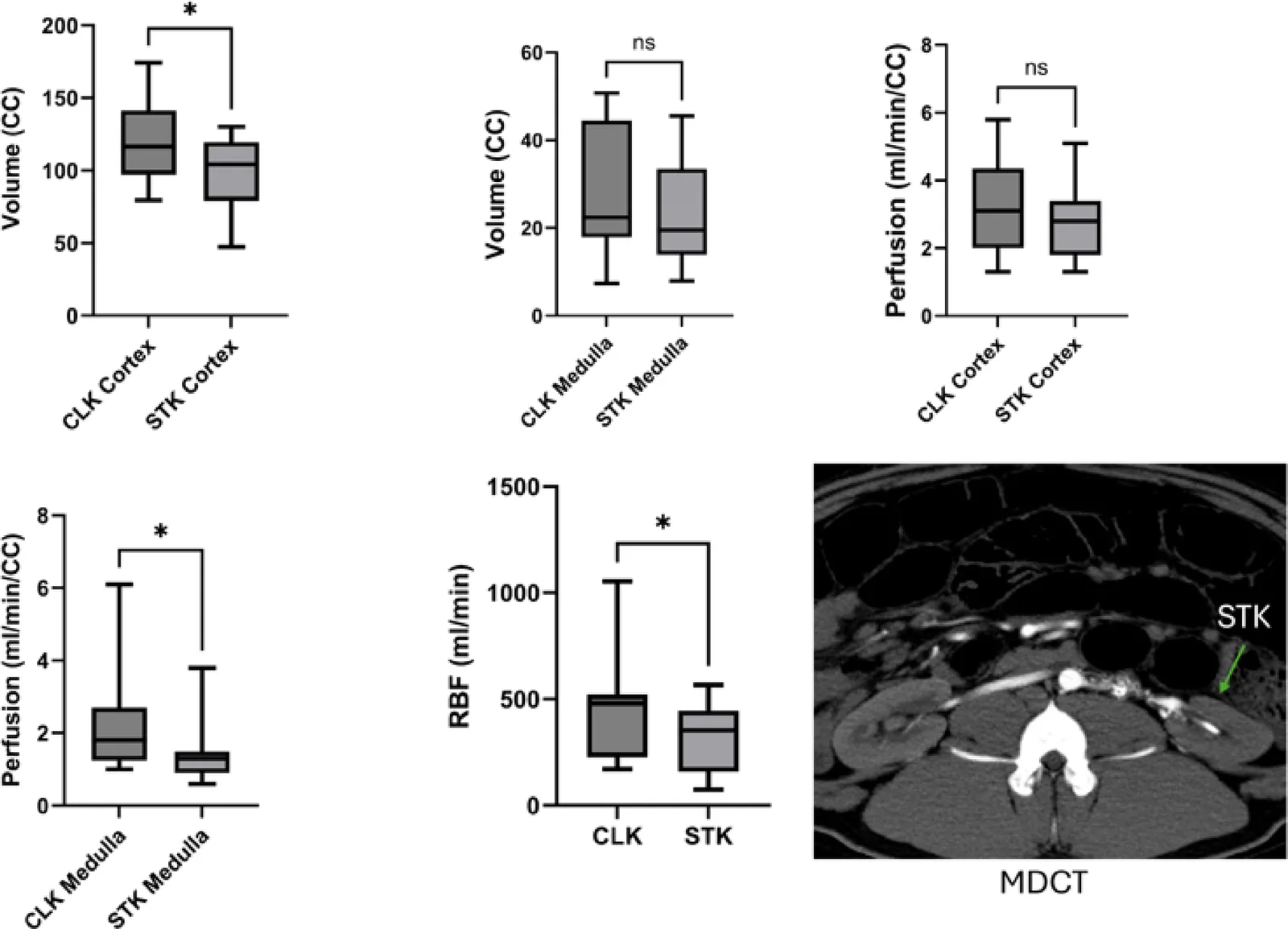
Multidetector computed tomography (MDCT) measurements of renal hemodynamics in renovascular disease (RVD) patients. Stenotic kidney (STK) cortical volume, medullary perfusion, and whole kidney renal blood flow (RBF) were lower compared to the contralateral (CLK), whereas STK medullary volume and cortical perfusion were not. Green arrow: STK. *p ≤ 0.05.

Cortical volume and medullary perfusion were lower in STK vs. CLK, as was STK renal blood flow (RBF), indicating a hemodynamically significant stenosis. However, STK medullary volume and cortical perfusion were similar to those observed in the CLK (Fig. 6).

### Associations between fibrogenic cytokines and MRI parameters

Although a paired comparison showed only a trend toward higher venous TIMP-1 levels in the STK compared to the CLK (*p* = 0.0708), TIMP-1 demonstrated significant direct associations with MRI markers of tissue remodeling. STK vein TIMP-1 levels correlated directly with cortical and medullary MTR-600 and − 1000 Hz and inversely with ADC, consistent with impaired extracellular matrix degradation and restricted water diffusion (Supplemental Fig. 1). STK vein pro-collagen-I level, reflecting active collagen synthesis, likewise showed significant direct correlations with cortical and medullary MTR-600 and − 1000 Hz at 3.0T (Supplemental Fig. 2). In contrast, collagen-IV, which reflects established extracellular matrix deposition, selectively associated with *f* at 1.5T in the cortex (*r* = 0.727, *p* = 0.007) and tended to associate with medullary *f*, but has not reached statistical significance (*r* = 0.500, *p* = 0.0609) (Supplemental Fig. 3).

### Levels of fibrogenic cytokines and miRNAs

There were no significant differences in plasma levels of pro-collagen-I, collagen-IV, or TIMP-1 among HV-2, RVD-CLK vein, STK vein (Supplemental Fig. 4A), and IVC; their urinary levels were also comparable (Supplemental Fig. 4B). Neither were there significant differences between levels of pro-fibrotic miR-150^16^, miR-214, and miR-21^17^, or anti-fibrotic miRNAs including miR-26a^18^ and miR-218^19^, in urinary exosomes of HV and RVD (Supplemental Fig. 5).

## Discussion

This study demonstrates that at 3.0T MRI both MTR-600 Hz and *f* can identify increased STK fibrosis in RVD compared to healthy kidneys. However, at 1.5T-MRI, MTR (at both 600 Hz and 1000 Hz frequencies) was also higher in the STK compared to the CLK, whereas cortical *f* only showed a nonsignificant trend toward higher values in the STK. Importantly, MTR and *f* values measured at 1.5T were similar to those obtained at 3.0T (except for STK-cortex at MTR-600 Hz), indicating that they are at least partially independent of the field-strength used. MDCT showed that RBF was reduced in the STK vs. CLK, consistent with a significant unilateral RVD, yet urinary and plasma indices of fibrotic factors were unchanged in these patients with minimally-impaired renal function. These observations support the use of MTI and qMT to detect imaging signatures consistent with mild fibrosis in post-stenotic kidneys.

RVD is a growing cause of CKD and ESRD, with renal fibrosis driving disease progression and irreversibility. PTRA and stenting are often ineffective due to persistent fibrosis and associated risks. Noninvasive methods for early fibrosis detection are urgently needed to guide targeted therapies.

In the absence of biopsies as a reference standard, we tested the ability of MTI and qMT to detect fibrosis indices in the STK of RVD patients against those in the CLK and healthy kidneys, as well as against levels of various fibrogenic biomarkers. We further compared the imaging-based fibrosis-indices obtained using 1.5T and 3.0T scanners to assess their machine-dependence and clinical potential.

Our study indicates that at 3.0T MRI, the development of STK fibrosis-related changes was detected by higher MTR-600 Hz and *f* values in RVD compared to both HV-1 and the CLK. Our previous studies demonstrated that STK-MTR-600 Hz acquired at 3.0T detects alterations occurring following PTRA in swine RVD and can noninvasively predict post-PTRA kidney fibrosis^20^. In contrast, although MTR-1000 Hz values were comparable between 1.5T and 3.0T-MRI in RVD patients, MTR-1000 Hz did not detect increased fibrosis compared to either HV-1 or the CLK. Interestingly, while MTR-600 Hz detected elevated imaging signatures consistent with STK fibrosis at 3.0T-MRI, it was not fully reproducible across the two field strengths, because STK-cortex at MTR-600 Hz differed between 1.5T and 3.0T. These findings highlight potential pitfalls of each MTI resonance frequency.

We also found that although qMT detected development of cortical and medullary fibrosis-related changes at 3.0T MRI, and its values were comparable to those obtained at 1.5T, at 1.5T *f* in the STK was not significantly different from the CLK. These findings suggest that while MTI and qMT are largely consistent across different machines and both detect changes consistent with STK fibrosis at 3.0T MRI, MTI outperforms qMT at 1.5T MRI.

MDCT identified lower RBF in the STK than in the CLK, consistent with the hemodynamically significant degree of STK stenosis^21^. Nonetheless, in contrast to MT methods, neither STK-BOLD (R2*) nor DWI (ADC and eADC) MRI differed between the STK and CLK, suggesting their limited utility in detecting mild fibrosis in RVD patients within our study setting. Given the relatively preserved eGFR in these patients, the extent of hypoxia and microstructural alterations were likely mild. This in turn highlights the ability of molecular MRI (e.g., MT methods) to detect subtle fibrosis-related remodeling in these patients.

Urinary or plasma levels of the pro-fibrogenic parameters collagen-IV and TIMP-1 have been used to assess development of renal fibrosis in several forms of renal disease^22–25^. TIMP-1^26^ and pro-collagen-I^27^ primarily reflect active fibrotic remodeling, encompassing increased matrix synthesis and reduced degradation, whereas collagen-IV^28^, an integral basement membrane component, more closely reflects established extracellular matrix deposition and structural remodeling, rather than dynamic fibrogenic activity per se. In support of the modest kidney fibrosis developing in our cohort, our RVD patients exhibited unaltered levels of these pro-fibrogenic parameters, even in plasma collected from the STK vein. Similarly, urinary fibrosis biomarkers (like TIMP1) are not elevated in diabetic children without microalbuminuria^29^. Nevertheless, RVD-STK TIMP-1 correlated directly with cortical and medullary MTR-600 Hz and − 1000 Hz, and inversely with ADC, and cortical and medullary MTR-600 Hz and − 1000 Hz correlated directly with plasma RVD-STK pro-collagen-I. On the other hand, cortical *f* directly correlated with plasma collagen-IV, suggesting that *f* may preferentially reflect established extracellular matrix deposition rather than dynamic fibrotic activity. Collectively, these findings suggest that kidney-specific biomarkers may reflect localized subtle fibrotic changes and may demonstrate context-specific relationships with distinct MRI-derived parameters. Notably, STK venous TIMP-1 levels correlated inversely with ADC, consistent with impaired water molecule motion in the fibrotic microenvironment.

We also tested whether levels of select anti-fibrogenic miRNAs (miR-26a and miR-218) were lower, or conversely whether pro-fibrogenic miRNAs (miR-214 and miR-150) were elevated, in RVD urinary exosomes compared to HV. Our previous study showed that renal vein levels of free microRNA-26a were reduced in the STK^18^. The miR-214 is markedly involved in interstitial fibrosis induced by unilateral ureteral obstruction^30^, and exosomal miR-150-5p derived from tubular cells promotes renal fibrosis following ischemia-reperfusion injury^31^, implicating them in renal fibrogenesis. However, the fibrotic and anti-fibrotic miRNAs cargo of urinary exosomes remained unchanged between the RVD and HV groups, again likely due to the modest changes in these kidneys.

It should be acknowledged that MT-MRI reflects alterations in macromolecular content and renal parenchymal microstructure as an indirect imaging biomarker. However, it does not provide a specific quantification of collagen deposition, nor does it enable discrimination among the various histological components of interstitial fibrosis. Furthermore, MT-MRI signals may be influenced by multiple confounders, including edema, inflammation, and vascular changes, which may limit its specificity for fibrosis assessment. Accordingly, although MT-MRI offers a non-invasive approach for evaluating renal structural alterations, its signal should not be interpreted as a direct quantitative surrogate for histopathological fibrosis severity. These limitations highlight the need for further development and validation of more specific imaging techniques for accurate assessment of interstitial fibrosis in patients with RVD.

### Limitations

Our study faced several limitations. Because renal biopsies were unavailable, fibrosis was inferred indirectly compared to the non-stenotic and healthy kidneys. The preserved renal function in RVD suggests likely modest tissue remodeling, yet both MT-MRI methods successfully detected subtle fibrosis-related changes in the STK. The comparison between 1.5T and 3.0T MRI was performed only in RVD patients. Furthermore, for logistical recruitment reasons the HVs included two different cohorts, restricting the ability to test direct comparisons of imaging and biological parameters obtained in this population. Urinary exosomes may not reflect fibrotic activity. The relatively small, predominantly white cohort (10 RVD patients and 22 HVs) may have limited the statistical power of the analyses, which could partly account for the marginal significance observed in some comparisons (e.g., *p = ~* 0.06), rather than absence of biological effect. Consequently, the possibility of type-II error cannot be excluded, and the robustness of certain findings may be affected. In addition, the limited cohort size may reduce the generalizability of the results. Therefore, these findings should be interpreted with caution. Future studies with larger, independent cohorts are needed to validate and further extend these observations. Lastly, MRI access, cost, and complexity may hinder clinical use.

## Conclusion

This study shows that at 3.0T-MRI both *f* or MTR-600 Hz may be useful noninvasive tools to assess mild kidney fibrosis in patients with RVD, whereas at 1.5T MTR-600 Hz more robustly detects elevated STK fibrosis compared to the CLK. On the other hand, *f* values are more faithfully reproduced between different magnetic field-strengths, because STK-cortex at MTR-600 Hz differed between 1.5T and 3.0T. Nevertheless, both MRI methods appear to be more sensitive in detecting RVD fibrosis than circulating or urinary fibrogenic markers. Since it can capture a full cross-section of the kidney, MT-MRI may provide better tissue representation with less sampling bias than renal biopsy, and can be safely repeated to monitor disease progression, prognosis, and treatment. Future studies should evaluate its consistency across different machines and its effectiveness in other etiologies of kidney fibrosis.

## Supplementary Information

Below is the link to the electronic supplementary material.

Supplementary Material 1

Supplementary Material 2

Supplementary Material 3

Supplementary Material 4

## Data Availability

The data that support the findings of this study are available from the corresponding author upon reasonable request.
