# Supplementary material for: MT-MRI for detection of renal interstitial fibrosis in renovascular disease
Source: Sci Rep. 2026 Jul 5;16:30834. doi: 10.1038/s41598-026-60712-6 (PMC13634916; doi:10.1038/s41598-026-60712-6)

Fig. 1

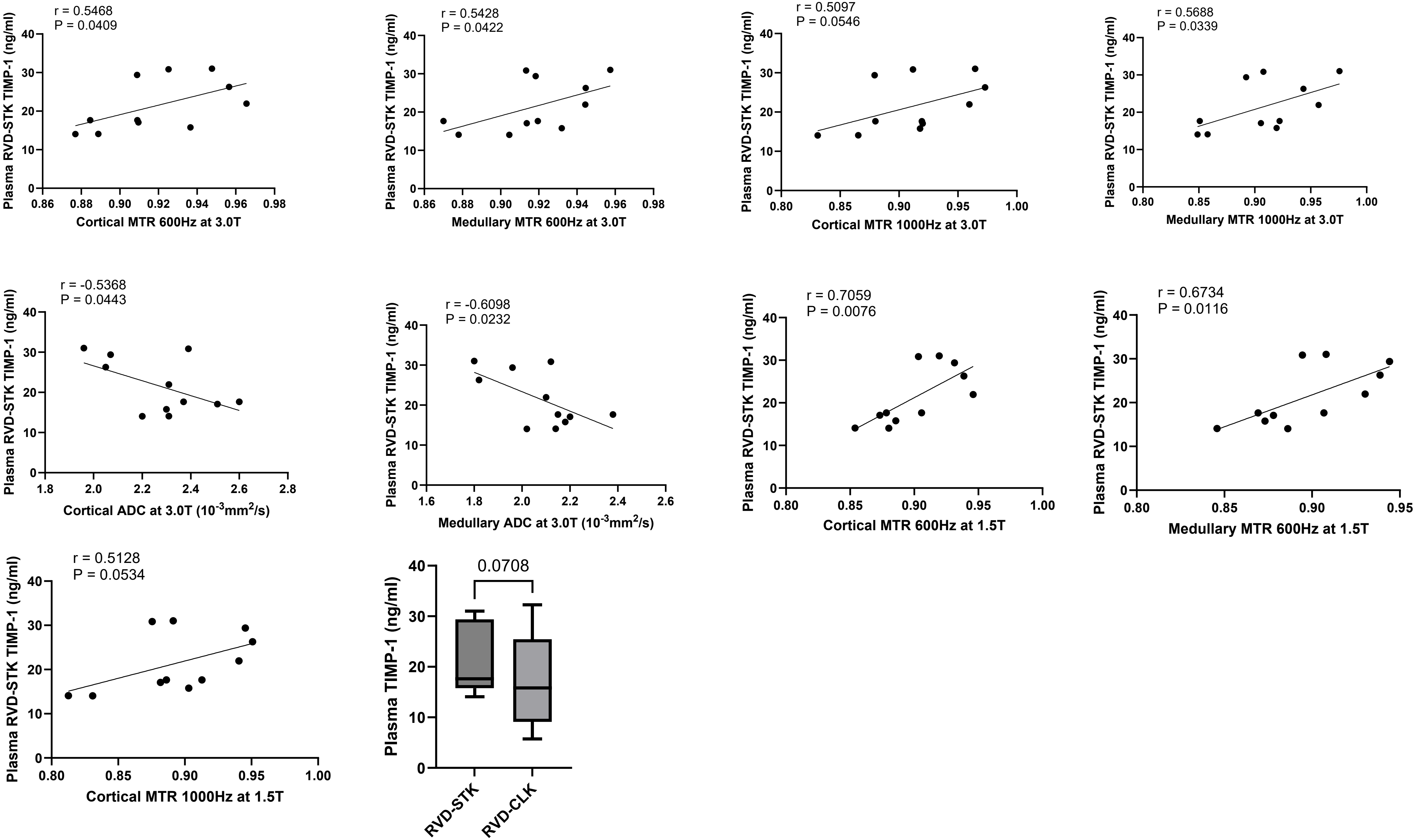
r = 0.5468
P = 0.0409
Plasma RVD-STK TIMP-1 (ng/ml)
Cortical MTR 600Hz at 3.0T
r = 0.5428
P = 0.0422
Plasma RVD-STK TIMP-1 (ng/ml)
Medullary MTR 600Hz at 3.0T
r = 0.5097
P = 0.0546
Plasma RVD-STK TIMP-1 (ng/ml)
Cortical MTR 1000Hz at 3.0T
r = 0.5688
P = 0.0339
Plasma RVD-STK TIMP-1 (ng/ml)
Medullary MTR 1000Hz at 3.0T
r = -0.5368
P = 0.0443
Plasma RVD-STK TIMP-1 (ng/ml)
Cortical ADC at 3.0T ($10^{-3}mm^2/s$)
r = -0.6098
P = 0.0232
Plasma RVD-STK TIMP-1 (ng/ml)
Medullary ADC at 3.0T ($10^{-3}mm^2/s$)
r = 0.7059
P = 0.0076
Plasma RVD-STK TIMP-1 (ng/ml)
Cortical MTR 600Hz at 1.5T
r = 0.6734
P = 0.0116
Plasma RVD-STK TIMP-1 (ng/ml)
Medullary MTR 600Hz at 1.5T
r = 0.5128
P = 0.0534
Plasma RVD-STK TIMP-1 (ng/ml)
Cortical MTR 1000Hz at 1.5T
0.0708
Plasma TIMP-1 (ng/ml)
RVD-STK
RVD-CLK

Fig. 2

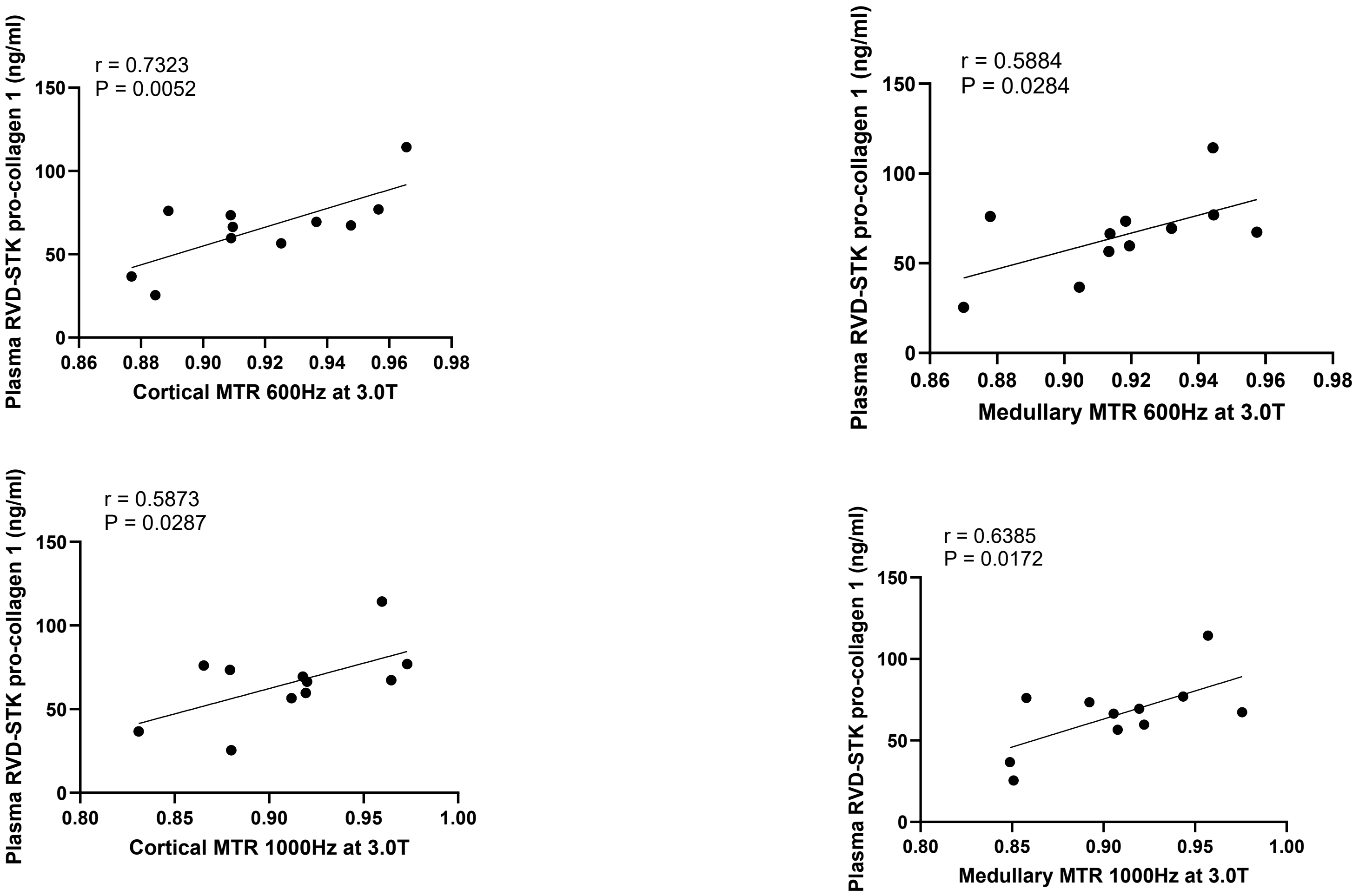

r = 0.7323
P = 0.0052
Plasma RVD-STK pro-collagen 1 (ng/ml)
Cortical MTR 600Hz at 3.0T
r = 0.5884
P = 0.0284
Plasma RVD-STK pro-collagen 1 (ng/ml)
Medullary MTR 600Hz at 3.0T
r = 0.5873
P = 0.0287
Plasma RVD-STK pro-collagen 1 (ng/ml)
Cortical MTR 1000Hz at 3.0T
r = 0.6385
P = 0.0172
Plasma RVD-STK pro-collagen 1 (ng/ml)
Medullary MTR 1000Hz at 3.0T

Fig. 3

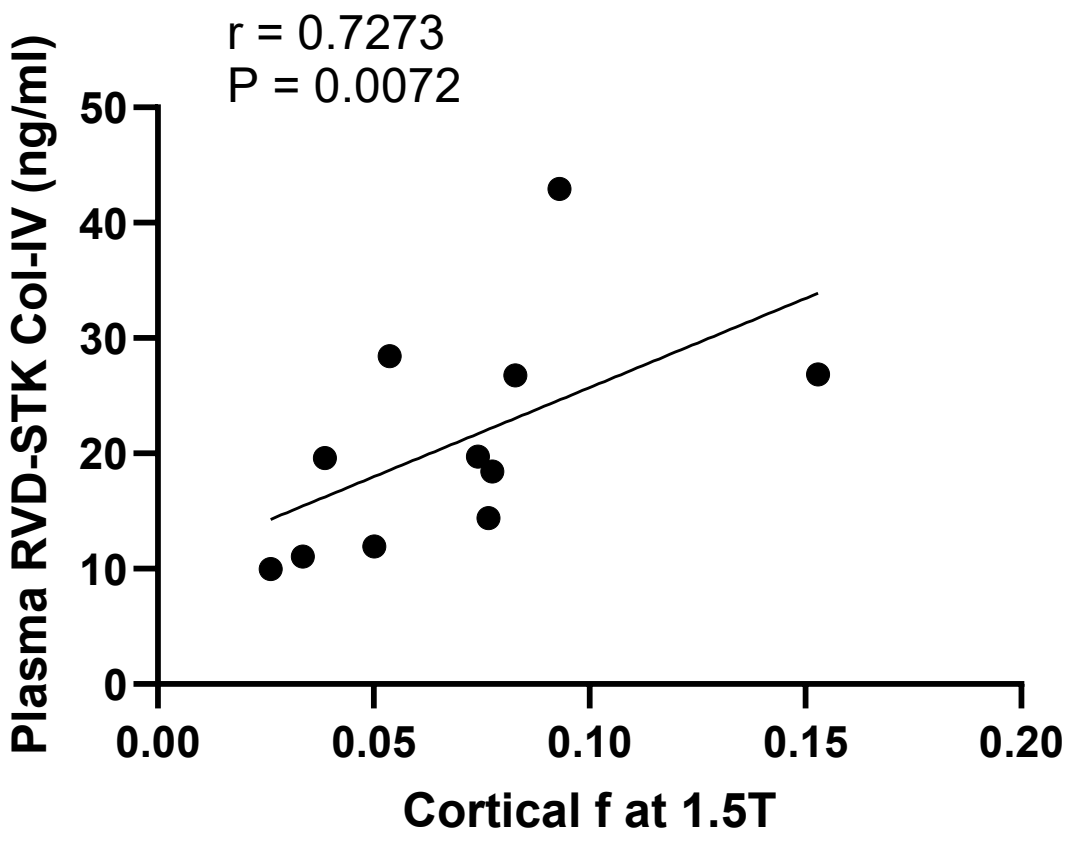

r = 0.7273
P = 0.0072
Plasma RVD-STK Col-IV (ng/ml)
50
40
30
20
10
0
0.00
0.05
0.10
0.15
0.20
Cortical f at 1.5T

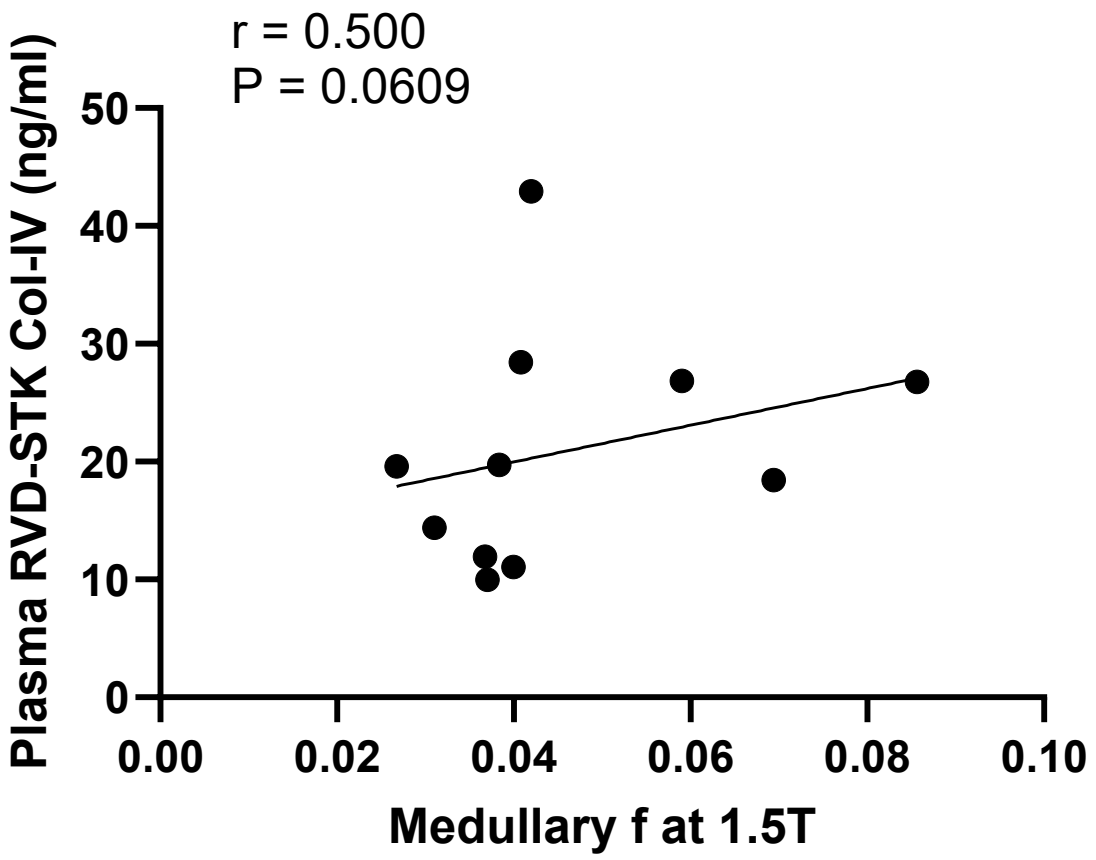

r = 0.500
P = 0.0609
Plasma RVD-STK Col-IV (ng/ml)
50
40
30
20
10
0
0.00
0.02
0.04
0.06
0.08
0.10
Medullary f at 1.5T

Fig. 4

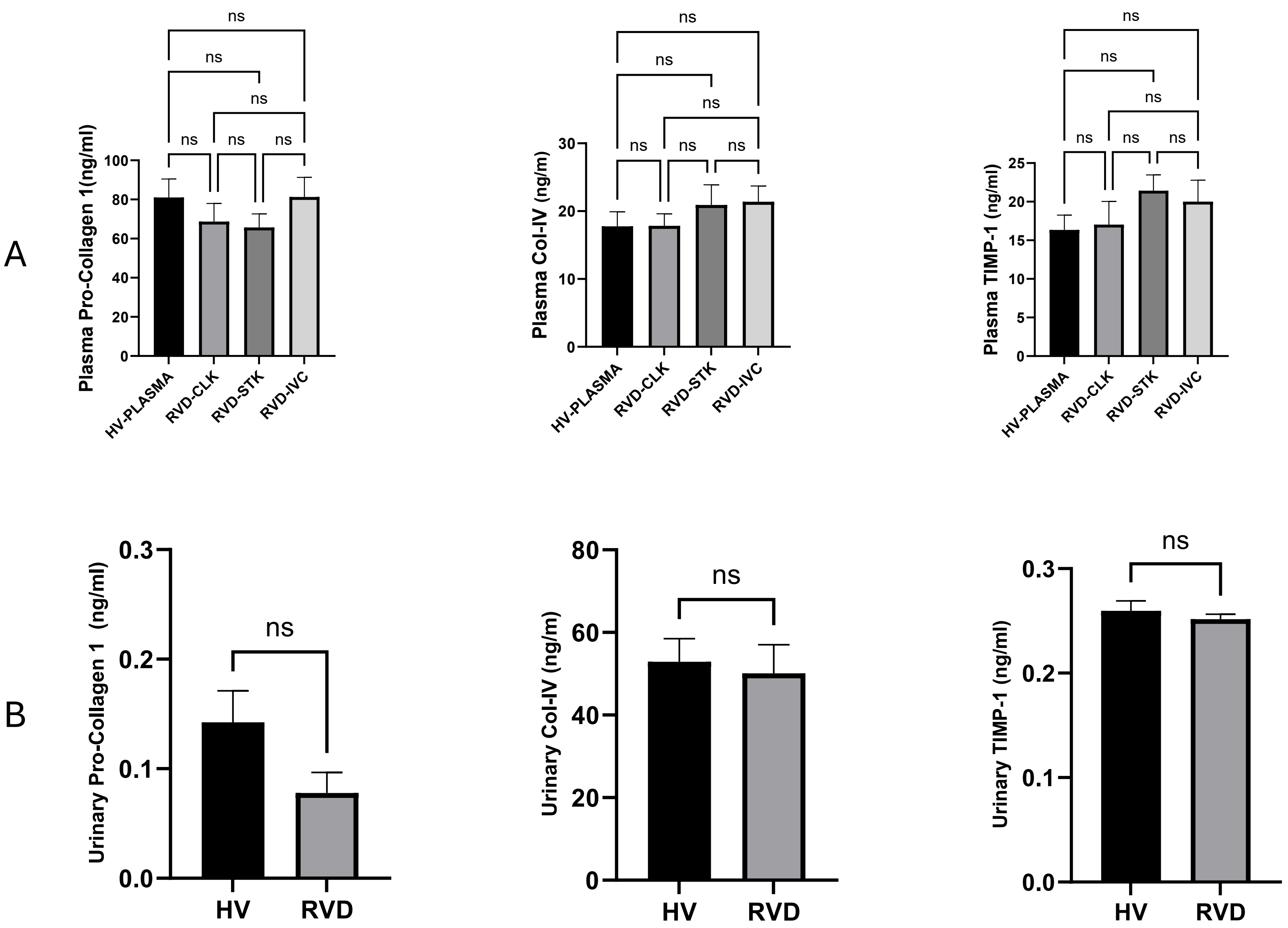

A
Plasma Pro-Collagen 1(ng/ml)
Plasma Col-IV (ng/m)
Plasma TIMP-1 (ng/ml)
HV-PLASMA
RVD-CLK
RVD-STK
RVD-IVC
ns
B
Urinary Pro-Collagen 1 (ng/ml)
Urinary Col-IV (ng/m)
Urinary TIMP-1 (ng/ml)
HV
RVD
ns

Fig. 5

Fibrogenic miRNAs

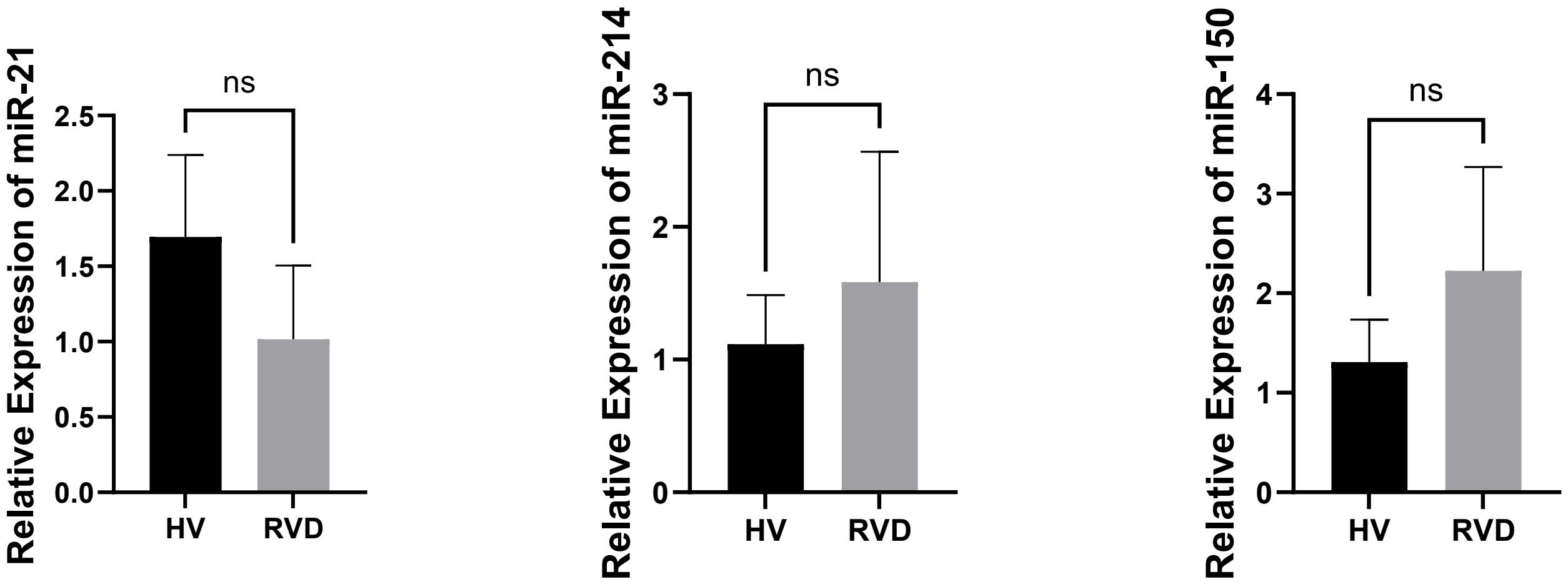

Anti-fibrogenic miRNAs

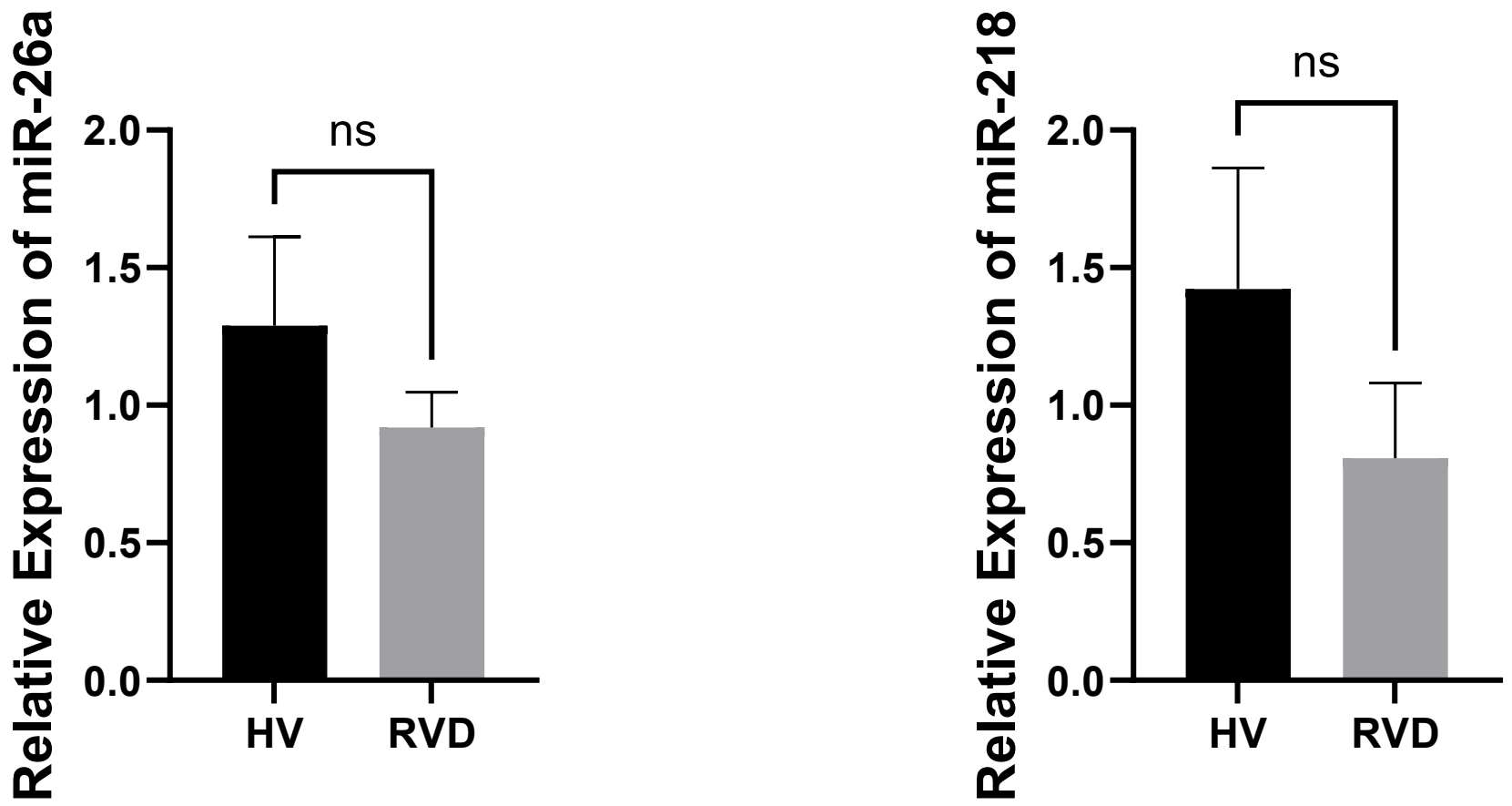

Supplement: Supplementary file 1 [file 41598_2026_60712_MOESM2_ESM.pdf]
