# Supplementary material for: MT-MRI for detection of renal interstitial fibrosis in renovascular disease
Source: Sci Rep. 2026 Jul 5;16:30834. doi: 10.1038/s41598-026-60712-6 (PMC13634916; doi:10.1038/s41598-026-60712-6)
Supplement: Supplementary file 2 [file 41598_2026_60712_MOESM1_ESM.docx]

Supplemental Figure 1. 1 (TIMP-1) with magnetization transfer ratio (MTR) and ADC. At 3.0T MRI, cortical and medullary MTR at 600Hz and 1000Hz were directly correlated with venous plasma levels of TIMP-1 in renovascular disease–stenotic kidney (RVD-STK), whereas apparent diffusion coefficient (ADC) showed an inverse correlation. At 1.5T MRI, medullary MTR at 600Hz and cortical MTR at 1000Hz also correlated with plasma RVD-STK TIMP-1. Yet, RVD-STK showed only a trend toward higher venous TIMP-1 levels compared with RVD–contralateral kidney (RVD-CLK).

Supplemental Figure 2. Pro-collagen I correlated with magnetization transfer ratio (MTR). At 3.0T, cortical and medullary magnetization transfer ratio (MTR) at 600 Hz and 1000 Hz correlated with plasma renovascular disease–stenotic kidney (RVD-STK) pro-collagen I.

Supplemental Figure 3. Collagen-IV correlated with *f*. At 1.5T, cortical and medullary *f* correlated with plasma renovascular disease–stenotic kidney (RVD-STK) collagen-IV.

Supplemental Figure 4. Fibrogenic factor levels in blood and urine of HV and RVD. A. Plasma pro-collagen-I alpha-1, collagen-IV, and tissue inhibitor of metalloproteinases-1 (TIMP-1) levels did not differ among healthy volunteers (HV), contralateral kidney (CLK), stenotic kidney (STK), and renovascular disease (RVD)-inferior vena cava (IVC). B. The urinary levels of these fibrogenic factors did not differ among these groups either. ns, not significant.

Supplemental Figure 5. Fibrosis-related and anti-fibrotic microRNAs in urinary exosomes of RVD patients and HV. Neither fibrosis-related micro-RNAs (miR-21, miR-214, and miR-150) nor anti-fibrotic micro-RNAs (miR-26a and miR-218) were different between the renovascular disease (RVD) and healthy volunteers (HV).
