# Supplementary material for: MT-MRI for detection of renal interstitial fibrosis in renovascular disease
Source: Sci Rep. 2026 Jul 5;16:30834. doi: 10.1038/s41598-026-60712-6 (PMC13634916; doi:10.1038/s41598-026-60712-6)
Supplement: Supplementary file 3 [file 41598_2026_60712_MOESM3_ESM.docx]

**Imaging procedures**

MTI, qMT, BOLD, and DWI non-contrast scans were performed in RVD and HV-1 with a 3.0T MRI Signa scanner (DISCOVERY MR750W, GE). In RVD patients , MTI and qMT were also acquired at a one-day interval using a 1.5T scanner (OPTIMA MR450W, GE, Waukesha, WI). ECG and blood pressure were continuously monitored throughout scanning.

**MTI**

MT and reference (M_0_) images were acquired in RVD using both 1.5T and 3.0T MRI scanners at offset frequencies of 600Hz and 1000Hz, both of which were previously shown to be sensitive to collagen-associated macromolecular signals while minimizing direct free-water saturation (6,7). The magnetization transfer ratio (MTR), representing the relative signal reduction in the MT image (acquired with magnetization transfer) compared to the baseline M_0_ image, serves as an MT-derived measure of kidney fibrosis. The MTR map for both the renal cortex and medulla was calculated pixel-wise as $\frac{M_{0}-MT}{M_{0}}$. To account for B1 inhomogeneity in both intrasubject and intersubject variations, the MTR values for each cortex and medulla were normalized using the muscle MTR, which was obtained by manually drawing a region of interest (ROI) in the dorsal muscle across all coronal slices. In HV-1, cortical and medullary MTR values for the cortex and medulla were obtained using only the 3.0T MRI.

**qMT-MRI**

B_0_ mapping was performed using the dual-echo time method, and B_1_ mapping using the Bloch-Siegert shift method. Two flip angles, 300° and 700°, were applied, with nine MT offset frequencies ranging from 1-50 kHz, resulting in a total of 18 MT scans. T_1_ kidney mapping utilized the saturation method along with adaptive recovery times (SMART1Map). The total acquisition time was approximately 4 min 20 sec excluding recovery periods; MT-weighted images at different offset frequencies were acquired during repeated end-expiratory breath-holds. Visual inspection revealed minimal inter-breath-hold motion. The full qMT scan therefore required several short breath-holds rather than a single continuous acquisition.

qMT parameters were derived using a classical two-pool exchange model describing magnetization exchange between a free-water pool and a macromolecular bound pool, based on the framework of Henkelman and Ramani. Voxel-wise nonlinear fitting of the MT-weighted data was performed using custom MATLAB scripts (MathWorks, Natick, MA). The 50-kHz offset image, which experiences minimal MT saturation, served as the reference signal (M₀). The main parameter of interest was the bound-pool fraction (*f*), an index of the extent of water-binding large molecules and a surrogate for renal fibrosis. Additional model parameters included the transverse relaxation times of the free and bound pools and the exchange rate term, which were estimated to support fitting. The apparent longitudinal relaxation rate of the free pool was obtained from the T₁ maps and incorporated into the model to derive the intrinsic longitudinal relaxation rate. The longitudinal relaxation rate of the bound pool was fixed at 1s⁻¹, consistent with prior studies. B₀ and B₁ maps were used to correct for local field inhomogeneities and determine the effective MT pulse frequency and power at each voxel. Cortical and medullary *f* values were extracted from manually defined ROIs. In RVD, qMT data from the 1.5T and 3.0T MRI scanners were compared to assess their reproducibility and field-strength- and scanner-dependence. For HV-1, cortical and medullary *f* values were obtained solely at 3.0T MRI.

**BOLD-MRI**

BOLD-MRI was utilized to evaluate cortical and medullary oxygenation at 3.0T-MRI. The acquisition of MR images with progressively increasing echo times enabled the computation of their regression with the signal logarithm, where the slope (R2*=1/T2*) was associated with deoxyhemoglobin concentrations and, by extension, blood and tissue oxygen levels. Image acquisition included coronal slices over both kidneys. A multi-echo gradient echo sequence captured T2*-weighted images at end-expiration, with a repetition time of 90 msec, and echo times ranging from 3.5-39.6msec, in 3.3msec intervals (12 images over 13sec).

**DWI**

DWI detects altered renal microstructures as the change in water molecule diffusivity. Maps of the DWI signal, apparent diffusion coefficient (ADC), were produced by fitting the MR signal intensity curve versus the corresponding b-values of 0, 30, 70, 100, 200, 400, and 800 s/mm² on a pixel-by-pixel basis to a mono-exponential decay model. A mono-exponential model was selected to maintain robustness and clinical feasibility given the relatively small cohort and scan-time considerations. Signal intensity at the highest b-value remained above the noise floor in all analyzed datasets. Cortical and medullary ROIs were outlined on the b0-DWI images obtained from 3.0T-MRI and transferred to the ADC maps. The mean ADC values were computed by averaging all the corresponding sampled ROIs. Furthermore, exponential ADC (eADC) maps, a qualitative measure with T2 shine-through that enhances cortical/medullary tissue contrast were derived from the same DWI dataset and evaluated similarly to ADC from b0 & DWI images.

**MDCT assessments of STK hemodynamics**

MDCT was performed only in RVD to assess bilateral, single-kidney, cortical and medullary renal blood flow (RBF), perfusion (mL/min/g), and volumes, as well as the degree of obstruction in the stenotic renal artery. We have developed and validated the MDCT method using renal time-density curves following contrast media injection.

In each CT image of the kidney, ROIs were marked in the cortex, medulla, and aorta on flow images to generate time-attenuation curves, which were analyzed using a custom Matlab module and used to assess cortical and medullary perfusion for each kidney (ml/min/cc tissue). Absolute blood flow (ml/min) was determined by multiplying values by their respective volumes, and RBF as the sum of cortical and medullary flows. The severity of stenosis was quantified from CT angiograms as the ratio between of the most narrowed and disease-free arterial cross-sectional areas.
