# Supplementary material for: MT-MRI for detection of renal interstitial fibrosis in renovascular disease
Source: Sci Rep. 2026 Jul 5;16:30834. doi: 10.1038/s41598-026-60712-6 (PMC13634916; doi:10.1038/s41598-026-60712-6)
Supplement: Supplementary file 4 [file 41598_2026_60712_MOESM4_ESM.docx]

Inclusion Criteria

1. Unilateral or bilateral atherosclerotic renovascular disease with at least one hemodynamically significant (>60% renal artery lumen occlusion)
2. Between ages 40 and 80 years old.
3. Patients with the diagnosis of chronic hypertension and/or requirement for at least one antihypertensive medication for more than 4 weeks.
4. Serum creatinine under 2.2 and 2.0 mg/dL for men and women, respectively (Caucasians). Values for African American subjects are slightly higher (2.4 mg/dL, males; 2.1 mg/dL females).
5. No contraindications to angiography: severe contrast allergy.
6. No contraindications to no-contrast MR evaluations: e.g., pacemaker or magnetically active metal fragments, claustrophobia.
7. Patients have the ability to comply with protocol
8. Patients are competent and able to provide written informed consent

Exclusion Criteria

1. Patient has serum creatinine >2.2 mg/dL for men and >2.0 mg/dL for women (Caucasians); >2.4 mg/dL for men and >2.1 mg/dL for women (African American).
2. RVD in a solitary kidney
3. Patients have clinically significant medical conditions within the prior six months: e.g. myocardial infarction, congestive heart failure, stroke, that would, in the opinion of the investigators, compromise the safety of the patient.
4. Uncontrolled hypertension (Systolic BP >180 mmHg despite therapy).
5. Uncontrolled diabetes or diabetes requiring insulin
6. Acute or chronic hepatitis B or C that requires treatment.
7. Evidence of HIV infection
8. Requirement for potentially nephrotoxic drugs, e.g., non-steroidal anti-inflammatory drugs.
9. Cardiac ejection fraction less than 30%.
10. History of deep venous thrombosis within 3 months of enrollment.
11. Kidney transplant.
12. Pacemaker, implantable defibrillator or other contraindication to MRI
13. Inability to comply with breath-hold for 20 seconds
14. Any active malignancy and undergoing therapy
15. Patients are pregnant.
16. Complicated and/or recurrent Kidney or ureteric stone(s) that require medical treatment or surgical intervention.
17. Another known acute or chronic kidney disease
18. Federal medical center inmates.
